# Population based prevalence of high blood pressure among adults in Addis Ababa: uncovering a silent epidemic

**DOI:** 10.1186/1471-2261-9-39

**Published:** 2009-08-23

**Authors:** Fikru Tesfaye, Peter Byass, Stig Wall

**Affiliations:** 1 School of Public Health, Addis Ababa University, Addis Ababa, Ethiopia; 2 Umeå International School of Public Health, Epidemiology and Public Health Sciences, Department of Public Health and Clinical Medicine, Umeå University, Umeå, Sweden

## Abstract

**Background:**

The prevention and control of high blood pressure or other cardiovascular diseases has not received due attention in many developing countries. This study aims to describe the epidemiology of high blood pressure among adults in Addis Ababa, so as to inform policy and lay the ground for surveillance interventions.

**Methods:**

Addis Ababa is the largest urban centre and national capital of Ethiopia, hosting about 25% of the urban population in the country. A probabilistic sample of adult males and females, 25–64 years of age residing in Addis Ababa city participated in structured interviews and physical measurements. We employed a population based, cross sectional survey, using the World Health Organization instrument for stepwise surveillance (STEPS) of chronic disease risk factors. Data on selected socio-demographic characteristics and lifestyle behaviours, including physical activity, as well as physical measurements such as weight, height, waist and hip circumference, and blood pressure were collected through standardized procedures. Multiple linear regression analysis was performed to estimate the coefficient of variability of blood pressure due to selected socio-demographic and behavioural characteristics, and physical measurements.

**Results:**

A total of 3713 adults participated in the study. About 20% of males and 38% of females were overweight (body-mass-index ≥ 25 kg/m^2^), with 10.8 (9.49, 12.11)% of the females being obese (body-mass-index ≥ 30 kg/m^2^). Similarly, 17% of the males and 31% of the females were classified as having low level of total physical activity. The age-adjusted prevalence (95% confidence interval) of high blood pressure, defined as systolic blood pressure (SBP) ≥ 140 mmHg (millimetres of mercury) or diastolic blood pressure (DBP) ≥ 90 mmHg or reported use of anti-hypertensive medication, was 31.5% (29.0, 33.9) among males and 28.9% (26.8, 30.9) among females.

**Conclusion:**

High blood pressure is widely prevalent in Addis Ababa and may represent a silent epidemic in this population. Overweight, obesity and physical inactivity are important determinants of high blood pressure. There is an urgent need for strategies and programmes to prevent and control high blood pressure, and promote healthy lifestyle behaviours primarily among the urban populations of Ethiopia.

## Background

Hypertension is an important public health problem worldwide. Analysis of the global burden of hypertension revealed that over 25% of the world's adult population had hypertension in 2000, and the proportion is expected to increase to 29% by 2025 [1]. According to the World Health Report 2002, cardiovascular disease accounted for 9.2% of total deaths in the African region in 2001 [2], and hypertension remains the most important risk factor with national prevalence levels ranging from 25% to 35% in adults aged 25–64 years [3]. Growing evidence suggests that hypertension constitutes the basis for the CVD epidemic in sub-Saharan Africa [4].

Limited data on the trends of prevalence of hypertension suggest that it has increased in economically developing countries in recent years while it remained stable or decreased in developed countries [5]. Thus, most of the world's hypertensive people live in developing countries, where cardiovascular disease has an early onset and higher mortality than in developed countries [6]. Studies have shown that hypertension is a strong contributor to the hazards of cardiovascular disease (CVD) in black Africans, with an odds ratio (OR) of 7.0 versus 2.3 to 3.9 in other ethnic groups, with P < 0.0002 [7].

The prevention and control of hypertension has not received due attention in many developing countries although it is one of the most modifiable risk factors of cardiovascular diseases. Awareness, treatment and control of hypertension is extremely low in these countries, as health care resources are overwhelmed by other priorities including HIV/AIDS, tuberculosis, and malaria.

Reliable epidemiological data are useful for the design and implementation of effective strategies for the prevention and control of hypertension. There are, however, limited data on the epidemiology of hypertension and its control in many sub-Saharan African countries. In this regard, the recent efforts by the World Health Organization (WHO) to generate comparable data on chronic disease risk factors in developing countries, using the stepwise surveillance (STEPS) instrument [8] are yielding useful information needed to build the empirical evidence base that should be accumulated in order to trigger the necessary policy response.

In this study, we report findings on the prevalence and some determinants of high blood pressure among adults in Addis Ababa, which were generated using the STEPS instrument. Addis Ababa represents the largest urban centre in Ethiopia, hosting about 25% of the urban population in the country. To date, Ethiopia has no national strategy for the prevention and control of chronic diseases or their risk factors. Findings of this study will, therefore, be useful to raise awareness among policy makers and the public at large, about the magnitude of high blood pressure and related risk factors of cardiovascular diseases, and thereby, contribute to the design and implementation of appropriate interventions.

## Methods

The present study, conducted in Addis Ababa City, constitutes part of a larger research project on chronic disease risk factors in Ethiopia. The city is organized into ten sub-cities, each of which are further divided into 9 or 10 administrative units known as 'kebeles', making up a total of 99 kebeles.

### Selection of study participants

This cross-sectional study employed a multi-stage sampling strategy, composed of random and cluster sampling techniques. Ten of the 99 kebeles were selected randomly, and smaller geographic units within the kebeles, known as zones or villages, were identified and listed for further random selection. Four zones were selected at random from each kebele, and the first household was randomly selected from each of the zones. Thereafter, subsequent households were selected on the basis of proximity to the first and the preceding household.

A probabilistic sample of 4001 adults in the age group 25–64 years were selected to participate in the study. Of these, 3713 (93%) gave informed and written (signed) consent, while the remaining participated in the study willingly but were unable to sign the consent form. Those who failed to sign the consent form were significantly more likely (P < 0.001) to be unable to read or write. In the present study, we report data from 3713 study participants who signed the consent form.

### Instruments and measurements

The WHO STEPS instrument [8] was used to collect data on selected socio-demographic characteristics and lifestyle behaviours including, physical activity, and physical measurements of weight, height, waist and hip circumference, as well as blood pressure. The scope of the present study is limited to behavioural and physical measurements, and does not include biochemical measurements.

Weight and height were measured with participants standing without shoes and wearing light clothing. Participants stood upright with the head in Frankfort plane for height measurement. Height was recorded to the nearest 0.5 cm, and weight was recorded to the nearest 100 g. Body-mass-index (BMI) was calculated as weight in kilograms over height in metres squared [weight (kg)/(height (m))^2^]. Waist circumference was measured at the level of the iliac crest using a non-elastic tape measure. Hip circumference was measured at the maximum circumference of the hip. Waist-to-hip ratio (WHR) was calculated as a ratio of waist and hip circumference.

The global physical activity questionnaire (GPAQ) section of the STEPS instrument was used for assessment of physical activity, and total physical activity was presented in MET (metabolic equivalent) minutes per week. The instrument looks into three major domains of day-to-day activities; work (including domestic work), transport, and recreational activities. Level of total physical activity was subsequently classified into high, moderate, or low using the GPAQ analysis guideline provided along with the STEPS instrument [8].

Blood pressure was measured in a sitting position using a digital device (**Omron *M4-I^®^***) after the study participant rested for at least five minutes. Three consecutive measurements were made in an interval of at least 3 minutes. Mean systolic and diastolic blood pressures were determined from the second and third measurements.

The questionnaire and physical measurement sections of the STEPS instrument have been previously validated and subsequently applied in predominantly rural settings in developing countries including Ethiopia [9,10].

Proportions and means were calculated with corresponding 95% confidence intervals (C.I) for descriptive findings, while multiple linear regression analysis was used to estimate the coefficient of variability of blood pressure due to selected socio-demographic and behavioural characteristics, as well as BMI and WHR.

### Ethical considerations

Ethical clearance was obtained from the Faculty of Medicine, Addis Ababa University. Informed and written (signed) consent was obtained from each study participant. Ethical conduct was maintained during data collection and throughout the research process.

## Results

### Description of the study population

A total of 3713 adults in the age group 25–64 years, from ten of the 99 kebeles (smallest administrative units) in Addis Ababa participated in the study. Median age of the study participants was 40 years, and 58.6% of them were females. The majority were Christians (93.1%), and from the Amhara (52.7%) or Oromo (20.2%) ethnic groups. About 23% had not had any formal education. Among the rest, the median duration of schooling was 9 years. About 72% of the males and 26% of the females were engaged in some income generating work through employment or self-employment (Table 1).

**Table 1:** Socio-demographic characteristics of the study participants, Addis Ababa, December 2006.

	Frequency	%
Sex		
Male	1538	41.4
Female	2175	58.6
Age group		
25–34	1117	30.1
35–44	1053	28.4
45–54	897	24.2
55–64	646	17.4
Religion		
Christian	3457	93.1
Moslem	256	6.9
Ethnic group		
Amhara	1957	52.7
Oromo	750	20.2
Guraghe	426	11.5
Tigre	311	8.4
Other	269	7.2
Education level		
No formal education	864	23.3
Primary (1–6)	843	22.7
Secondary (7–12)	1566	42.2
Above secondary	440	11.9
Paid work (income generating)		
Yes	1668	44.9
No	2045	55.1

### Behavioural risk factors

About 13.5% of the males and less than 1% of the females reported 'current' cigarette smoking, while 11% of the males were 'current daily' smokers. Significantly higher (P < 0.001) proportion of males (18.6%) than females (2.1%) reported current khat (*Cata edulis *Forsk) chewing. Close to 16% of males chewed khat one or more days per week. Infrequent heavy alcohol intake, defined as consuming five or more standard units of alcohol per day on one or two days during the week prior to interview was significantly higher (P < 0.001) in males (10.4%) than in females (0.9%).

Fresh leaves and buds of the khat plant are habitually chewed to attain a state of stimulation, energy, alertness, and to facilitate social interaction. Khat chewing is commonly accompanied by cigarette smoking. The khat leaves contain cathinone and cathine, which are responsible for the amphetamine-like effects of khat. We reported the prevalence of khat chewing and smoking in an earlier study (9).

### Salt intake

More males (12.5%) than females (9.4%) reported that they add salt to their plate after food has been served. Overall, 10.7%, 95% CI (9.7, 11.6) adults reported regularly adding salt to their plate.

### Body-mass-index (BMI)

Weight and height measurements were conducted in all but 10 (0.3%) individuals. Mean (95% CI) BMI was 22.25 (22.08, 22.42) among males and 24.07 (23.87, 24.27) among females. The majority had a normal BMI, while 20.2% of males and 37.7% of females were overweight (BMI ≥ 25). The prevalence of overweight was significantly higher in females than in males (P < 0.001), and it increased with increasing age group (Chi-square = 72.3, P < 0.001). The prevalence of obesity (BMI ≥ 30) was significantly higher in females (10.8%) than males (2.0%) (Chi-square = 95.8, P < 0.001). On the other hand, significantly more males (12.8%) than females (9.4%) were underweight (BMI < 18.5) (Chi-square = 8.9, P < 0.01). (Tables 2 and 3)

**Table 2:** Age- and sex-specific distribution of mean (95% CI) body mass index (BMI), total physical activity, systolic and diastolic blood pressure among adults in Addis Ababa.

	**Male**		**Female**	
	**Mean**	**95% CI**	**Mean**	**95% CI**

Body-mass-index (BMI, mean kg/m^2^) by age group				
25–34	21.63	(21.37, 21.85)	22.81	(22.48, 23.14)
35–44	22.30	(21.99, 22.61)	24.36	(23.98, 24.74)
45–54	22.72	(22.33, 23.11)	24.59	(24.19, 24.99)
55–64	22.98	(22.47, 23.49)	24.66	(24.18, 25.14)
*25–64*	*22.25*	*(22.08, 22.42)*	*24.07*	*(23.87, 24.27)*
				
Total physical activity (MET* minutes per week) by age group				
25–34	8717	(7882, 9553)	5748	(5018, 6477)
35–44	8098	(7187, 9010)	4927	(4368, 5486)
45–54	8794	(7608, 9980)	4812	(4249, 6180)
55–64	5214	(4249, 6180)	2676	(2231, 3121)
*25–64*	*7967*	*(7480, 8454)*	*4709*	*(4414, 5005)*
				
Systolic blood pressure (SBP, mean mmHg) by age group				
25–34	123.7	(122.5, 124.9)	116.9	(115.5, 118.3)
35–44	124.7	(123.1, 126.3)	122.4	(120.6, 124.2)
45–54	131.7	(129.2, 134.1)	130.0	(128.0, 132.0)
55–64	147.4	(144.0, 150.8)	140.1	(137.4, 142.7)
*25–64*	*129.4*	*(128.4, 130.5)*	*126.2*	*(125.1, 127.2)*
				
Diastolic blood pressure (DBP, mean mmHg) by age group				
25–34	78.5	(77.4, 79.5)	77.7	(76.7, 78.8)
35–44	80.6	(79.5, 81.7)	79.4	(78.3, 80.5)
45–54	83.2	(81.7, 84.7)	81.6	(80.5, 82.7)
55–64	86.0	(84.3, 87.6)	82.0	(80.5, 83.4)
*25–64*	*81.2*	*(80.6, 81.9)*	*80.0*	*(79.5, 80.6)*

Dec 2006.

*MET = metabolic equivalent; CI: confidence interval

**Table 3:** Distribution of body mass index (BMI) categories, overweight and level of total physical activity among adults in Addis Ababa, December 2006.

	**Male (n = 1535)**		**Female (n = 2175)**	
	**%**	**(95% CI)**	**%**	**(95% CI)**

BMI category (kg/m^2^)				
<18.5	12.8	(11.13, 14.47)	9.4	(8.17, 10.63)
18.5–24.99	66.9	(64.92, 68.88)	52.9	(50.8, 55.0)
25.0–29.99	18.2	(16.27, 20.13)	26.9	(25.0, 28.77)
≥ 30	2.0	(1.30, 2.70)	10.8	(9.49, 12.11)
				
Overweight (BMI ≥ 25) by age group				
25–34	14.5	(12.7, 16.2)	25.4	(23.5, 26.2)
35–44	20.0	(18.0, 22.0)	40.8	(38.7, 42.8)
45–54	25.3	(23.1, 27.4)	42.0	(39.9, 44.0)
55–64	26.9	(24.6, 29.1)	43.8	(41.7, 45.8)
*25–64*	*20.2*	*(18.1, 22.2)*	*37.7*	*(35.6, 39.7)*
				
Abdominal obesity*	12.9	(11.2, 14.5)	64.6	(62.59, 66.61)
				
Level of total physical activity				
High (vigorous)	51.6	(49.1, 54.1)	36.0	(33.98, 38.02)
Moderate	31.5	(29.1, 33.8)	32.8	(30.83, 34.77)
Low (sedentary)	16.9	(15.0, 18.7)	31.2	(29.25, 33.15)

*Abdominal obesity: waist-to-hip ratio > 1 in men, and waist-to-hip ratio > 0.85 in women.

CI: confidence interval

### Waist-to-hip ratio (WHR)

The mean (SD) WHR was 0.92 (0.19) in males and 0.88 (0.09) in females. About 13% of the males and 6.7% of the females had a WHR exceeding 1. Applying a separate cut-off point for WHR in females at >0.85, the proportion with high WHR (abdominal obesity) would be significantly (P < 0.001) higher in females (64.6%) than in males (12.9%). In 16 (0.4%) individuals, waist or hip measurements could not be taken due to lack of cooperation (Table 3).

### Physical activity

The average total physical activity (TPA, in MET-minutes per week) was estimated to be 7967 in males and 4709 in females. About 52% of the males and 36% of the females were classified as having a high (vigorous) level of TPA. On the other hand, significantly more females (31.2%) than males (16.9%) were classified as having low level of TPA or being sedentary (Chi-square = 88.6, P < 0.001) (Tables 2 and 3).

### Blood pressure

Three consecutive measurements of blood pressure were taken from 3273 (88.1%) of the study participants. The remaining individuals refused to participate in the second or third measurement of blood pressure, mainly due to the discomfort that repeated measurements created. Those who declined for repeated measurements were significantly (P < 0.01) more likely to be females and Christians compared to their counter parts. A systematic decline was observed across the three consecutive measurements, such that the second measurement was lower than the first, and the third was lower than the second. Consequently, mean systolic and diastolic blood pressure levels were significantly higher among the participants on whom three measurements were not obtained, compared to those who participated in all three repeated measurements. We used the average of the second and third measurements in all the analyses related to blood pressure.

Mean (95% CI) SBP was 129.4 mmHg (128.4, 130.5) among males and 126.2 mmHg (125.1, 127.2) among females. Similarly, mean (95% CI) DBP was 81.2 mmHg (80.6, 81.9) among males and 80.0 mmHg (79.5, 80.6) among females. Both mean SBP (P < 0.001) and DBP (P < 0.01) were significantly higher in males than in females. Overall, 31.5% of the males and 28.9% of the females had high blood pressure (defined as SBP/DBP ≥ 140/90 or reported use of anti-hypertensive medication). There was no significant difference between males and females in the prevalence of high blood pressure (P > 0.05). Among the 991 participants identified as having high blood pressure, 348 (35.2%) were aware of their blood pressure, but only 109 (11%) were receiving anti-hypertensive medication, of which 28 (25.6%) had normal (controlled) blood pressure at the time of the study.

Considering the differences in age composition between the male and female study participants, adjusted means and prevalence of high blood pressure were calculated. Thus, the age adjusted mean SBP (mmHg) in males and females were 129.9 and 125.5, respectively. The age adjusted mean DBP (mmHg) in males and females were 81.5 and 79.8, respectively. Similarly, the age-adjusted prevalence of high blood pressure was 32.5% in males and 27.9% in females.

The prevalence (95% CI) of isolated systolic hypertension was 8.9% (7.4, 10.3) in males and 8.3% (7.0, 9.5) in females. Isolated diastolic hypertension was 5.8% (4.5, 7.0) in males and 6.0% (4.9, 7.0) in females. Combined systolic and diastolic hypertension was 16.0% (14.0, 17.9) in males and 13.6% (12.0, 15.1) in females (Table 4).

**Table 4:** Prevalence of high blood pressure (HBP*) and isolated forms of hypertension across age groups and sex, among adults in Addis Ababa, December 2006.

	**Male (n = 1398)**		**Female (n = 1875)**	
	**%**	**(95% CI)**	**%**	**(95% CI)**

High blood pressure (HBP*) by age group				
25–34	19.8	(16.3, 23.2)	14.5	(11.3, 17.6)
35–44	24.6	(20.3, 28.8)	22.9	(19.3, 26.4)
45–54	36.5	(30.6, 42.3)	34.4	(30.3, 38.4)
55–64	63.2	(56.9, 69.4)	51.2	(45.7, 56.6)
*25–64*	*31.5*	*(29.0, 33.9)*	*28.9*	*(26.8, 30.9)*
				
Isolated systolic hypertension^1 ^by age group				
25–34	5.1	(3.1, 7.0)	2.1	(0.8, 3.3)
35–44	6.8	(4.3, 9.2)	3.5	(1.9, 5.0)
45–54	7.6	(4.4, 10.8)	9.3	(6.8, 11.7)
55–64	22.5	(17.1, 27.8)	24.2	(19.5, 28.8)
*25–64*	*8.9*	*(7.4, 10.3)*	*8.3*	*(7.0,9.5)*
				
Isolated diastolic hypertension^2 ^by age group				
25–34	6.7	(4.5, 8.8)	6.6	(4.3, 8.8)
35–44	6.8	(4.3, 9.2)	7.4	(5.1, 9.6)
45–54	6.5	(3.5, 9.4)	4.9	(3.0, 6.7)
55–64	1.3	(0.1, 2.7)	4.3	(2.0, 6.5)
*25–64*	*5.8*	*(4.5, 7.0)*	*6.0*	*(4.9,7.0)*
				
Systolic and diastolic hypertension^3 ^by age group				
25–34	7.5	(5.2, 9.7)	5.2	(3.2, 7.1)
35–44	11.1	(8.0, 14.1)	11.8	(9.0, 14.5)
45–54	21.7	(16.7, 26.6)	18.5	(15.1, 21.8)
55–64	36.8	(30.5, 43.0)	21.1	(16.6, 25.5)
*25–64*	*16.0*	*(14.0, 17.9)*	*13.6*	*(12.0, 15.1)*

	**Male (n = 440)**		**Female (n = 541)**	

Isolated systolic hypertension^1 ^by age group, in those with HBP*				
25–34	26.0	(21.9, 30.1)	14.3	(11.3, 17.2)
35–44	27.6	(23.4, 31.7)	15.3	(15.2, 18.3)
45–54	20.8	(17.0, 24.5)	26.9	(23.1, 30.6)
55–64	35.6	(31.1, 40.0)	47.3	(43.0, 51.5)
*25–64*	*28.4*	*(24.1, 32.6)*	28.8	*(24.9, 32.6)*
				
Isolated diastolic hypertension^2 ^by age group, in those with HBP*				
25–34	34.0	(29.5, 38.4)	45.7	(41.5, 49.9)
35–44	27.6	(23.4, 31.7)	32.3	(28.3, 36.2)
45–54	17.7	(14.1, 21.2)	14.3	(11.3, 17.2)
55–64	2.1	(0.7, 3.4)	8.5	(6.1, 10.8)
*25–64*	18.4	*(14.7, 22.0)*	20.7	*(17.2, 24.1)*
				
Systolic and diastolic hypertension^3 ^by age group, in those with HBP*				
25–34	38.0	(33.4, 42.5)	35.7	(31.6, 39.7)
35–44	44.9	(40.2, 49.5)	51.6	(47.3, 55.8)
45–54	59.4	(54.8, 63.9)	53.8	(49.6, 58.0)
55–64	58.2	(53.5, 62.8)	41.2	(37.0, 45.3)
*25–64*	50.9	*(46.2, 55.5)*	47.1	*(42.8, 51.3)*

*High blood pressure (HBP): systolic blood pressure ≥ 140 mmHg or diastolic blood pressure ≥ 90 mmHg, or if a person is on anti-hypertensive treatment,

^1^Isolated systolic hypertension: systolic blood pressure ≥ 140 mmHg and diastolic blood pressure <90 mmHg,

^2^Isolated diastolic hypertension: systolic blood pressure < 140 mmHg and diastolic blood pressure ≥ 90 mmHg,

^3^Systolic and diastolic hypertension: systolic blood pressure ≥ 140 mmHg and diastolic blood pressure ≥ 90 mmHg.

Among adults with high blood pressure (n = 981), 28.4% (95% CI, 24.1, 32.6) of males and 28.8% (95% CI, 24.9, 32.6) of females had isolated systolic hypertension; and 18.4% (95% CI, 14.7, 22.0) of males and 20.7% (95% CI, 17.2, 24.1) of females had isolated diastolic hypertension. The prevalence (95% CI) of systolic and diastolic hypertension in this subgroup of adults was 50.9% (46.2, 55.5) in males and 47.1% (42.8, 51.3) in females (Table 4).

### Regression analysis

Multiple linear regression analysis identified sex to be the strongest predictor of mean SBP (β = 7.4 mmHg) and DBP (β = 2.5 mmHg), (P < 0.001), which led us to stratify subsequent analyses by sex. Age and BMI were significantly associated (P < 0.001) with mean SBP and DBP in both males and females, while educational level was inversely associated with both SBP and DBP in males. Waist-to-hip ratio was significantly (P < 0.05) associated with both mean SBP and DBP in females. Current daily smoking was associated with SBP, while level of total physical activity was inversely association with SBP in males (Tables 5 and 6).

**Table 5:** Beta-coefficient (β) for systolic blood pressure among adults in Addis Ababa, Dec 2006.

	**Males (n = 1398)**				**Females (n = 1875)**			
	**β**	**SE**	**P-value**	**95% CI for β**	**β**	**SE**	**P-value**	**95% CI for β**

Age	0.60	0.04	0.000	(0.50, 0.68)	0.56	0.05	0.000	(0.45, 0.66)
Education*	-0.45	0.11	0.000	(-0.66, -0.22)	-0.48	0.11	0.000	(-0.69, -0.25)
Religion	1.23	1.79	0.495	(-2.30, 4.75)	3.92	1.93	0.043	(0.12, 7.72)
Ethnic group	-0.41	0.50	0.418	(-1.39, 0.58)	0.21	0.53	0.692	(-0.83, 1.26)
Body mass index	1.48	0.15	0.000	(1.18, 1.78)	0.88	0.10	0.000	(0.66, 1.09)
Waist-to-hip ratio	0.70	2.44	0.775	(-4.10, 5.50)	12.17	5.67	0.032	(1.04, 23.20)
Total physical activity	-1.64	0.65	0.013	(-2.92, -0.35)	0.37	0.58	0.528	(-0.78, 1.52)
Current daily smoking	2.26	1.61	0.161	(-0.90, 5.42)	-9.37	14.53	0.519	(-37.8, 19.12)
Adding salt on plate	-0.32	1.46	0.828	(-3.19, 2.55)	-2.41	1.70	0.158	(-5.75, 0.93)
Binge drinking	-1.08	1.65	0.516	(-4.33, 2.17)	4.33	4.99	0.386	(-5.46, 14.12)

* Education refers to the highest grade completed

SE: standard error; CI: confidence interval

**Table 6:** Beta-coefficient (β) for diastolic blood pressure among adults in Addis Ababa, Dec 2006.

	**Males (n = 1398)**				**Females (n = 1875)**			
	**β**	**SE**	**P-value**	**95% CI for β**	**β**	**SE**	**P-value**	**95% CI for β**

Age	0.21	0.02	0.000	(0.14, 0.26)	0.09	0.03	0.005	(0.02, 0.14)
Education*	-0.05	0.07	0.513	(-0.18, 0.09)	-0.07	0.06	0.263	(-0.20, 0.05)
Religion	-0.00	1.13	0.997	(-2.23, 2.22)	0.49	1.13	0.666	(-1.73, 2.70)
Ethnic group	-0.23	0.31	0.471	(-0.85, 0.39)	0.02	0.31	0.952	(-0.59, 0.63)
Body mass index	0.95	0.09	0.000	(0.76, 1.13)	0.74	0.06	0.000	(0.61, 0.86)
Waist-to-hip ratio	0.29	1.54	0.853	(-2.72, 3.32)	6.62	3.31	0.046	(0.11, 13.12)
Total physical activity	-0.63	0.41	0.130	(-1.44, 0.18)	-0.16	0.34	0.636	(-0.83, 0.51)
Current daily smoking	3.26	1.01	0.001	(1.25, 5.25)	-10.5	8.49	0.215	(-27.19, 6.12)
Adding salt on plate	-0.83	0.92	0.372	(-2.64, 0.99)	-1.36	0.99	0.171	(-3.13, 0.59)
Binge drinking	1.34	1.04	0.200	(-0.71, 3.39)	-0.55	2.91	0.852	(-6.26, 5.17)

* Education refers to the highest grade completed

SE: standard error; CI: confidence interval

## Discussion

The assessment of some cardiovascular disease risk factors was conducted using the STEPS instrument for the first time in Addis Ababa, as a continuation of a similar study in a semi-urban part of the country, Butajira [10]. Data on behavioural and lifestyle related risk factors on a sample of urban and rural adults in Ethiopia are also available from the 2003 Ethiopia National Health Survey (2003 ENHS) that was conducted as part of the World Health Survey [11]. However, the 2003 ENHS did not include anthropometric or blood pressure measurements, and measurement of physical activity was based on the international physical activity questionnaire (IPAQ) [12], while we used the GPAQ.

A few other surveys reported data on blood pressure and BMI. One of these included young people aged 15–24 in Addis Ababa in 1994 [13], while another included a rural sample of adults in 1983. The comparability of these studies to ours is limited due to differences in age, residence, and survey instruments, among others.

Our study revealed widespread prevalence of various risk factors of cardiovascular diseases among adults in Addis Ababa. Overweight and obesity, including abdominal obesity, were more prevalent among females, while elevated blood pressure was high in both males and females. A quarter of the adult population has sedentary lifestyle. Urbanization influences are apparent in the city, with an increasing use of motorized transport and sedentary types of occupation such as trade and office work. This is accompanied by shifting dietary and lifestyle behaviours, which are yet to be characterized adequately.

Our findings revealed a considerably high prevalence of overweight among adults in Addis. Few studies reported BMI levels from population-based measurements in Ethiopia. One notable study is the 2005 Ethiopia Demographic and Health Survey (EDHS) [14] which indicated that women in Addis had the highest prevalence of overweight across the country. However, the level (17.5%) was much lower than our estimate of 37.7%. Mean BMI in females was 24.0 in our study sample and 22 in the EDHS [14]. Differences in age composition and sample size could account for much of the differences in the estimates between the two studies. We enrolled a representative sample of 2175 females in the age group 25–64, while the EDHS included 325 women 15–49 years of age in Addis. Furthermore, it is unclear whether the EDHS sample could be representative at the regional level.

Both studies revealed that BMI increased with increasing age. Thus, the wider age group and the consequently higher mean age of our study participants might have contributed to the higher overall prevalence of overweight in Addis. On the other hand, age-specific levels of overweight are markedly different between the two studies. We believe that the larger sample size and wider age representation of our study offers a more accurate estimate for prevalence of overweight among women in Addis.

Our findings in Addis contrast sharply with the situation in Butajira, where data were generated using the same methodology and instrument (STEPS) from a sample composed of urban and rural populations [10]. Mean BMI was 19.4 in males and 19.2 in females 25–64 years of age in Butajira. The proportion with BMI ≥ 25 was 2.5% and 2.2% among males and females, respectively. BMI increased significantly with increasing age in the Addis population, while there was no such association in the Butajira population [10].

More comparable findings were reported from other sub-Saharan African countries that applied the STEPS methodology and instruments recently. According to the WHO Global InfoBase [15], the prevalence of overweight in men was 30% in Ghana, 17% in The Sudan, and 15% in the United Republic of Tanzania. Similarly, the prevalence of overweight in women was reported to be about 28% in Ghana, 29% in The Sudan, 27% in the United Republic of Tanzania, 22% in Kenya, and 22% in Uganda. Our findings are slightly higher than the figures cited for some African countries and comparable to the others. However, it has to be noted that the reports for the other countries are national estimates, which include both urban and rural populations while we reported figures for the urban population in Addis Ababa. It can be assumed that the prevalence in urban centres of the countries cited here might be comparable or even higher than that in Addis. More appropriate comparisons could be made by making use of age-, sex -, and residence-specific prevalence figures.

It is evident from the findings that the prevalence of overweight among adults in Addis is exceeding that of underweight or chronic energy deficiency. The large contrast between urban (overweight) and rural (underweight) populations signifies the rising burden of overweight in urban populations, and the double burden that countries like Ethiopia are faced with.

Our estimate of the level of physical inactivity (25.3%) is higher than the global estimate, which ranges between 11% and 24% among adults 15 years and above [2], while moderate physical activity (32.3%) is on the lower side of the global estimate that ranged from 31% to 51%, with a global average of 41%. The ENHS [11] also reported 16.3% prevalence of physical inactivity among urban adults. Some of the variability between these reports could be due to the differences in the methods or instruments used to assess physical activity.

The mean SBP among adults in Addis was higher than their counterparts in Butajira, which were 117.2 mmHg in males and 108.8 mmHg in females. The mean DBP was similarly lower in Butajira, 75.3 mmHg and 70.7 mmHg in males and females, respectively. The prevalence of high blood pressure was reported to be 12.3% in males and 8.3% in females in Butajira [10]. There is an apparent gradient of urbanization between the two populations that in turn determines the distribution of behavioural, dietary and intermediate risk factors of cardiovascular diseases. Although the measurements were conducted using similar methods and instruments in the two populations, a consistent gradient has emerged in blood pressure, BMI, level of physical activity and other risk factors across the two populations, signalling that the urban areas might be the epicentre of the growing epidemic of chronic diseases and their risk factors in the country. Higher prevalence of hypertension in urban than rural sub-populations was also reported within the Butajira study [10] study. Similar findings on increasing blood pressure with increasing level of urbanization were reported by a study in South Africa [16], and throughout sub-Saharan Africa [17].

Data on mean blood pressure levels in Ethiopia and other African countries are provided online by the WHO Global InfoBase [15]. The reported figures reveal comparable values of mean SBP for men and women in Ethiopia, Ghana, Uganda, and The United Republic of Tanzania, and higher values for the Sudan. These national estimates are lower than the levels we report for the Addis population, for the apparent reason that national averages are diluted by lower values from rural populations.

The age-adjusted prevalence of hypertension revealed a gender gap (32.5% in males, 27.9% in females) that is similar to other studies in Africa. Edwards et al. [18] also reported a prevalence of 30% in men and 26.6% in women in Tanzania in 2000. A wider gender dichotomy was reported more commonly from Caucasian populations [19], while some other studies have also revealed higher prevalence of hypertension among women than men [20].

Both the mean SBP and DBP were significantly higher in males than females in Addis Ababa. A similar finding has been reported in Nigeria [21]. Linear association between blood pressure and age has been widely reported [22]. In our study population, significant linear association was demonstrated between age and SBP as well as DBP, which was stronger with SBP; β = 0.59, 95% CI (0.50, 0.68) in males and β = 0.56; 95% CI (0.45, 0.66) in females, than with DBP; β = 0.20, 95% CI (0.14, 0.26) in males and β = 0.08, 95% CI (0.02, 0.14) in females.

SBP decreased by about 0.45 mmHg in males and 0.48 mmHg in females for every additional year of schooling completed in our study population. Inverse relationship between education and blood pressure has been widely reported in studies from economically developed countries [23-28]

The INTERMAP study that included 4680 men and women aged 40–59 years from 17 diverse population samples in Japan, People's Republic of China, UK, and USA, demonstrated a strong significant inverse relationship between education and blood pressure, which was more manifest in the USA participants [29]. The same study suggested that the inverse association between education and blood pressure might be due to confounding by multiple dietary factors and BMI that accounted for the adverse blood pressure levels in less educated than more educated Americans.

The INTERSALT study that involved 52 population samples in 32 countries worldwide, reported the inverse relationship in women for 38 samples and in men for 28 samples [30]. In the same study, the large part of the inverse association was accounted for by BMI, smoking, alcohol intake, and 24-hour urinary sodium and potassium excretion [31,32].

Our findings reveal that mean SBP and DBP levels in both sexes were sub-optimal (greater than 120 and/80 mmHg, respectively). The prevalence of elevated blood pressure is a cause for concern in Ethiopia as in other African countries. The WHO African Regional Office [3] reported that prevalence of hypertension ranges from 25% to 35% in adults aged 25–64 years in some African countries that conducted the WHO STEPS survey.

We demonstrated a significant association between BMI and blood pressure in both sexes, which was consistent across mean SBP and DBP. A study in Tanzania [32] reported a positive and independent association of SBP and DBP with BMI and with age. Other studies have also reported that BMI is positively and significantly associated with blood pressure across populations in diverse geographic settings [33-36]. The continuous and linear relationship between BMI and blood pressure signifies the importance of BMI as a determinant of blood pressure in the Addis population.

Salt intake has been difficult to measure accurately or reliably at a population level. We were limited by resource constraints and the scope of the study to measure the 24-hour sodium excretion, which might have yielded a more quantitative account of the sodium balance. Thus we employed a qualitative approach in order to describe dietary salt use behaviour, by inquiring about 'adding salt on the plate' after food has been served (during a meal). However, this approach could not detect those people who might have modified their behaviour due to awareness of their high blood pressure. This failure to establish a temporal relationship between salt intake behaviour and blood pressure, due to the cross-sectional design of the study, might have contributed to the lack of association between 'adding salt on the plate' and blood pressure in our study population. On the other hand, as salt use behaviour may be changing along with increasing urbanization and modernization, a qualitative assessment of salt use behaviour may still be valuable for the purpose of surveillance.

### Limitations

As the scope of the present study does not include biochemical measurements, we were unable to determine the level of blood glucose or cholesterol. Similarly, we have not collected data on menopausal status among the women, and thus we have not controlled the potential effect of menopausal status on the risk of blood pressure in women.

## Conclusion

The prevalence of high blood pressure among adults in Addis Ababa is a cause for concern, as it constitutes a silent epidemic that is not matched with comparable level of awareness among policy makers or intervention by the health system. Considering the fact that there is only a limited pool of information on the burden of CVD or their risk factors in Ethiopia, an important input of this study would be to raise awareness about the problem among the population and policy makers so as to bring chronic diseases in general, and hypertension in particular, into the health research and policy agenda. Programs for the prevention and control of cardiovascular diseases and their risk factors should be designed and implemented as a matter of urgency.

## Competing interests

The authors declare that they have no competing interests.

## Authors' contributions

FT designed the study, developed survey instrument, supervised data collection and data entry, analyzed data and wrote manuscript. PB and SW participated in the study design, supervised instrument development, reviewed analysis and contributed to manuscript editing.

## Pre-publication history

The pre-publication history for this paper can be accessed here:


